# The application of a 4D-printed chitosan-based stem cell carrier for the repair of corneal alkali burns

**DOI:** 10.1186/s13287-024-03653-z

**Published:** 2024-02-14

**Authors:** Zibo Wang, Changqing Jiang, Yuqiao Fan, Xiaodan Hao, Yanhan Dong, Xinjia He, Jinning Gao, Yongchun Zhang, Meng Li, Mengyuan Wang, Yiming Liu, Wenhua Xu

**Affiliations:** 1 Institute of Regenerative Medicine and Laboratory Technology Innovation, Qingdao University, Qingdao, 266071 Shandong China; 2 Department of Clinical Laboratory, Affiliated Hospital of Qingdao University, Qingdao, 266003 China; 3 Department of Pathology, Qingdao Municipal Hospital, Qingdao, 266000 Shandong China; 4 Institute of Translational Medicine, College of Medicine, Qingdao University, Qingdao, 266003 Shandong China; 5 Department of Oncology, Affiliated Hospital of Qingdao University, Qingdao, 266003 Shandong China

**Keywords:** Chitosan, 4D printing technology, Cell scaffold, Limbal epithelium stem cells, Corneal wound healing

## Abstract

**Background:**

Corneal alkali burns can lead to ulceration, perforation, and even corneal blindness due to epithelial defects and extensive cell necrosis, resulting in poor healing outcomes. Previous studies have found that chitosan-based in situ hydrogel loaded with limbal epithelium stem cells (LESCs) has a certain reparative effect on corneal alkali burns. However, the inconsistent pore sizes of the carriers and low cell loading rates have resulted in suboptimal repair outcomes. In this study, 4D bioprinting technology was used to prepare a chitosan-based thermosensitive gel carrier (4D-CTH) with uniform pore size and adjustable shape to improve the transfer capacity of LESCs.

**Methods:**

Prepare solutions of chitosan acetate, carboxymethyl chitosan, and β-glycerophosphate sodium at specific concentrations, and mix them in certain proportions to create a pore-size uniform scaffold using 4D bioprinting technology. Extract and culture rat LESCs (rLESCs) in vitro, perform immunofluorescence experiments to observe the positivity rate of deltaNp63 cells for cell identification. Conduct a series of experiments to validate the cell compatibility of 4D-CTH, including CCK-8 assay to assess cell toxicity, scratch assay to evaluate the effect of 4D-CTH on rLESCs migration, and Calcein-AM/PI cell staining experiment to examine the impact of 4D-CTH on rLESCs proliferation and morphology. Establish a severe alkali burn model in rat corneas, transplant rLESCs onto the injured cornea using 4D-CTH, periodically observe corneal opacity and neovascularization using a slit lamp, and evaluate epithelial healing by fluorescein sodium staining. Assess the therapeutic effect 4D-CTH-loaded rLESCs on corneal alkali burn through histological evaluation of corneal tissue paraffin sections stained with hematoxylin and eosin, as well as immunofluorescence staining of frozen sections.

**Results:**

Using the 4D-CTH, rLESCs were transferred to the alkali burn wounds of rats. Compared with the traditional treatment group (chitosan in situ hydrogel encapsulating rLESCs), the 4D-CTH-rLESC group had significantly higher repair efficiency of corneal injury, such as lower corneal opacity score (1.2 ± 0.4472 vs 0.4 ± 0.5477, *p* < 0.05) and neovascularization score (5.5 ± 1.118 vs 2.6 ± 0.9618, *p* < 0.01), and significantly higher corneal epithelial wound healing rate (72.09 ± 3.568% vs 86.60 ± 5.004%, *p* < 0.01).

**Conclusion:**

In summary, the corneas of the 4D-CTH-rLESC treatment group were similar to the normal corneas and had a complete corneal structure. These findings suggested that LESCs encapsulated by 4D-CTH significantly accelerated corneal wound healing after alkali burn and can be considered as a rapid and effective method for treating epithelial defects.

**Graphical abstract:**

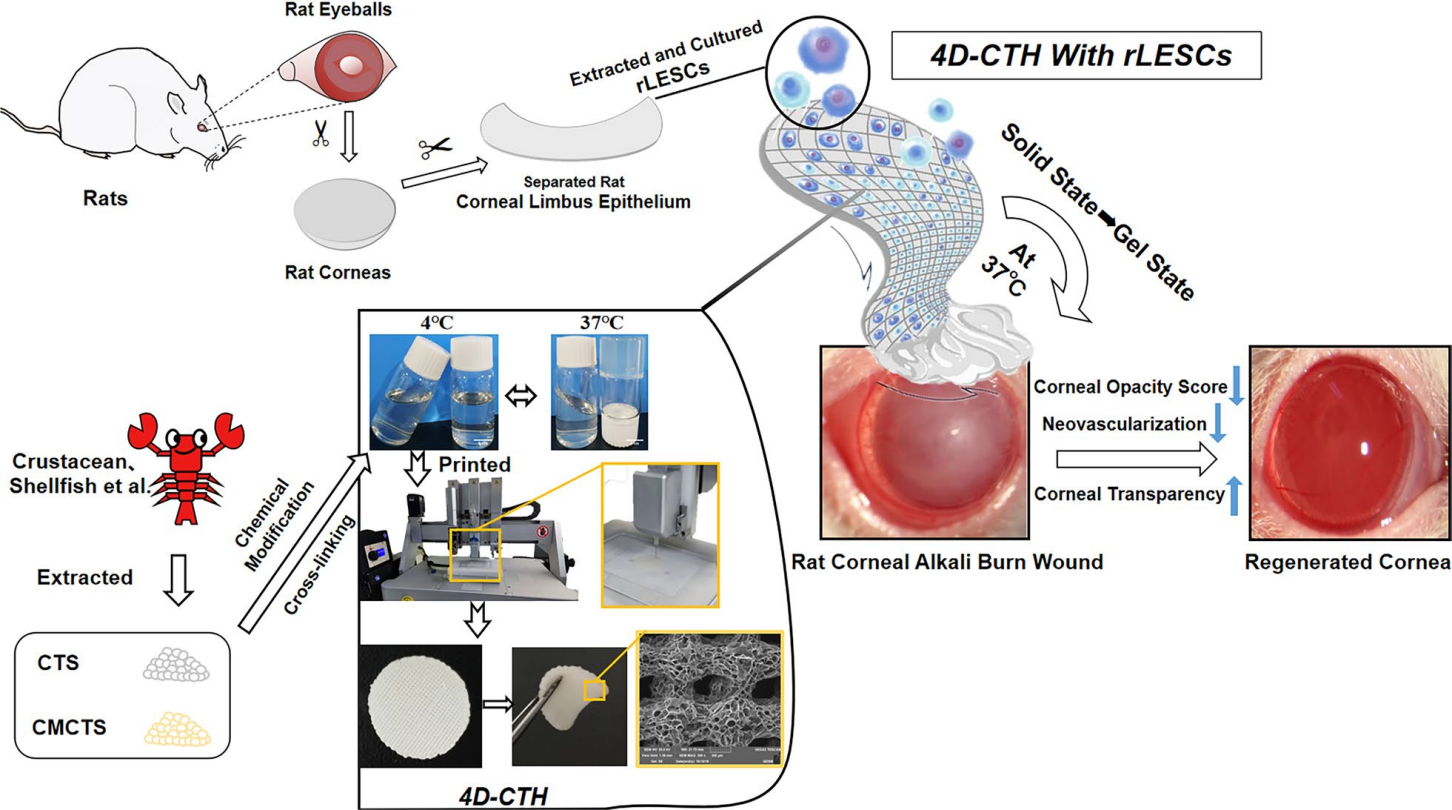

**Supplementary Information:**

The online version contains supplementary material available at 10.1186/s13287-024-03653-z.

## Introduction

Corneal alkali burns are serious chemical injuries where the local micro- environment is destroyed due to alkali saponification, causing corneal epithelial necrosis, exfoliation, corneal self-melting, and perforation [2, 20, 56]. Moreover, due to the formation of numerous new capillaries, corneal blindness can occur in severe cases [52]. Conventional corneal alkali burn treatment has focused on promoting the reconstruction of the damaged epithelium, alleviating inflammation, and preventing complications such as corneal lysis and scar formation [1, 10]. The amniotic membrane has been used as a major substitute for corneal transplantation in recent years [33]. However, as a basement membrane, it is easy to curl and be degraded by collagenase, which seriously affects healing [64]. Therefore, how to repairing corneal epithelial defects is still an urgent clinical problem that needs resolution.

Limbal epitheliumstem cells (LESCs) are a special type of epithelial cell located in the limbal basal epithelium and an important source of corneal epithelium regeneration [9]. The treatment of severe corneal injuries must begin with two processes: one is to provide seed cells for the timely repair of the corneal epithelium, and the other is to improve the microenvironment for cell growth [47, 53]. Previous reports have shown that the survival rate of LESCs will be effectively improved by tissue engineering vector [15, 47, 58]. Therefore, the development of safe and reliable cell scaffolds for LESCs transplantation is critical.

Our previous study utilized the direct cross-linking properties of sodium alginate oxide and carboxymethyl chitosan to form a heat-sensitive gel and transfer the carrier to the surface of keratopathy using a double syringe, providing a local growth microenvironment of LESCs and promoting the repair of the injury [67]. However, through scanning electron microscopy observations, we found that the surface of the prepared alginate-chitosan hydrogel (ACH) had an irregular network structure with different pore sizes, which directly reduced the loading efficiency of the LESCs. Therefore, How to improve the transfer rate and survival rate of stem cells is an urgent problem to be solved in severe corneal injury [29, 31, 39].

Four-dimensional (4D) printing technology is based on three-dimensional (3D) printing technology to add a time dimension [8, 63, 68]. 4D bioprinting can leverage shape memory capabilities and 3D bioprinting strategies to change the shape or behavior of biological materials [35, 37]. 4D bioprinting also benefits from the properties of smart materials, creating scaffolds that can mimic the structural properties of normal tissues [61]. These carriers offer good cytocompatibility and low toxicity, which can greatly improve the encapsulation rate of cells and the microenvironment for cell growth.

In this study, we will use two kinds of polysaccharide derivatives and 4D bioprinting technology to prepare a novel stem cell carrier, which could have uniform pore size and adjustable shape, and then a serious of experiments will be carried out to prove if LESCs’ loading rate and repair effect will be improved through this carrier on cornea damage.

## Materials and methods

### Materials and reagents

All animal experiments were conducted in strict accordance with the National Institute of Health guidelines for the care and use of laboratory animals. Male SD (Sprague Dawley) rats were purchased from Beijing Sibeifu Experimental Animal Science and Technology Co., Ltd. (Beijing, China), Chitosan (degree of deacetylation ≥ 95%, viscosity 100–200 mpa s) was purchased from Shanghai Maclean Biochemical Co., Ltd. (Shanghai, China), Carboxymethyl Chitosan (CMCTS, degree of deacetylation = 96.44%, 134 kDa) was prepared in our laboratory [67], and β-glycerophosphoric disodium were purchased from Shanghai Soraby Biotechnology Co., Ltd. Human corneal epithelial cells (immortalized monkey virus 40 HCECs) were purchased from the Institute of Biochemistry and Cell Biology, Chinese Academy of Sciences (Shanghai, China). A Cell Counting Kit-8 was purchased from Anhui Bishop Co., Ltd., China. Anti-cytokeratin 3 antibody (AB68260), anti-p40-DeltaNp63 (AB203826), Rabbit IgG (AB172730), goat anti-rabbit IgG H&L (Alexa Fluor®488) (AB150077), and goat anti-mouse IgG H&L (Alexa Fluor®594) (AB150116) were obtained from Abcam (Cambridge, UK). Anti-cytokeratin 12 antibodies (SC-515882, Delaware, USA) were provided by Santa Cruz Biotechnology, anti-cytokeratin 13 antibodies (A0411) were purchased from Abclonal (Wuhan, China), and anti-vimentin antibodies (AF7013) were purchased from Affinity Biosciences (Ohio, USA). Pentobarbital sodium was purchased from Sigma-Aldrich (Missouri, USA). Laminin-521 (LN521) was purchased from BioLamina (Sundbyberg, Sweden). Neutral protease (Dispase II) and cyclosporin A (CyA) was purchased from Solarbio (BeiJing, China). Calcein-AM/PI solution (MA0361) was purchased from Meilunbio (Dalian, China). Dulbecco modified Eagle medium/F12 medium (DMEM/F12), fetal bovine serum (FBS), trypsin, penicillin, and streptomycin were purchased from Gibco (Grand Island, NY, USA).

### Preparation of the 4D-CTH scaffold material

Under aseptic conditions, 0.1–0.3 g of chitosan was dissolved in 4.5 mL of 0.1 mmol/L of acetic acid solution and stirred for 10 h. Then the solution was mixed with 2.2–6.7% carboxymethyl chitosan and 6–8% β sodium glycerophosphate to form a new mixed solution which we named CTH, and CTH was printed into a homogeneous carrier with a pore size of 200 microns using 4D printing technology at a low temperature (Bio-architect®SR, Regenovo, China). Then, the materials were freeze-dried in a vacuum and used as carrier scaffolds for cell transplantation.

### Physical behavior of the 4D-CTH scaffold material

The 4D-CTH sheets were freeze-dried in a vacuum, and then the products were sprayed with gold, and observed and photographed using a scanning electron microscope (SEM) (Tescan, Brno, Czech Republic). The freeze-dried scaffold materials were placed in double distilled water until swelling equilibrium was reached and the weight no longer increased. Then, the swelling ratio of the scaffold materials was analyzed. The hydrogel was fabricated into slices 10 mm in diameter and 1 mm thick, which were then placed on the support plate of the rheometer. The storage modulus and loss modulus of the gel slices were measured at a shear frequency of 0.1–100 Hz and strain of 1%. Then, the hydrogel structure was analyzed by a superconducting nuclear magnetic resonance spectrometer (Bruker AVANCE III HD 400 MHz, Germany). The chitosan-based temperature-sensitive hydrogel was dissolved in D_2_O at 4 °C for 12 h in the dark to form a colorless and transparent liquid, which was injected into the nuclear magnetic tube using a micro-sampler for 1 h of nuclear magnetic testing.

The ATR-FTIR (Attenuated Total Reflection Flourier transformed Infrared Spectroscopy) spectra were recorded on a Nicolet^TM^iS50 FTIR spectrometer (Thermo Scientific, Waltham, MA, USA), covering a frequency range from 400 to 4000 cm^−1^. The 4D-CTH samples were frozen and lyophilized in a freeze dryer, and then all of the studied samples were prepared in KBr. Data analysis and drawings were carried out by OriginPro 9.1.

### Cytocompatibility of the 4D-CTH with rLESCs

#### Primary culturing and identification of the rLESCs

The rat limbal epithelial stem cells (rLESCs) were obtained from the eyes of ten healthy adult SD rats. The rats were euthanized by cervical dislocation, and the entire eyeballs were removed, washed with phosphate buffer solution (PBS) several times, and soaked in 400 U/mL of penicillin or streptomycin for half an hour. Remove the 1 mm-sized round tissue at the junction of conjunctiva and corneal gray matter under the microscope, cut it into uniform tissue pieces, and place it in DMEM/F12-dissolved 2 U/mL Disase II. For digestion overnight at 4 °C. After digestion, attach the corneal epithelial layer separated from the stromal layer to the LN521-coated (0.8 μg/cm^2^) culture flask [36]. Then, cell migration was observed daily under an inverted microscope.

Primary rLESCs and human corneal epithelial cells were cultured on culture plate slides for five days and identified by immunofluorescence staining, the antibody dilution for CK3 and CK12 was both 1:100 [67]. Then, the images were observed and collected using a fluorescent microscope (Olympus, Tokyo, Japan).

The phenotype of the rLESCs at the third generation (P3) was detected by flow cytometric analysis. To identify the expression of the intracellular marker deltaNp63 in the rLESCs, the cells were detached with 0.25% trypsin solution (without EDTA) and collected, and washed with PBS buffer containing 5% BSA. Then, the cells were fixed with 4% paraformaldehyde, and 0.5% triton × 100 was added to break down the membrane. Subsequently, the cell suspension was incubated with rabbit monoclonal anti-p40-DeltaNp63 (Abcam, AB203826) and goat anti-rabbit IgG H&L (Alexa Fluor®488). The working concentration of anti-p40-DeltaNp63 was 1:400. Finally, the cells were washed twice with PBS buffer and flow cytometry was performed using Cyto FLEXS (Beckman Coulter). We repeated the experiment three times and analyzed three groups of extracted rLESCs. The antibodies and cell concentrations used in the control and experimental groups were the same for each experiment. The gating thresholds for the three groups of cells were set at 10,000, 3000, and 3000, and the analysis software used was FlowJo version 10.4.

#### Detection of cytocompatibility

The cytocompatibility of 4D-CTH with rLESCs was evaluated by Cell Counting Kit-8 (CCK-8) cell proliferation assay, live/dead cell staining, and cell scratch assay. The rLESCs were inoculated in the 96-well plates (200 μL/well) and the 6-well plates, incubated at 37 °C for 24 h. The culture medium was replaced with 4D-CTH extract for the experiment group, which was a cell culture medium soaked for 7 days with CTH and containing 0.2 g/mL dried scaffold powder. Cells cultured in medium alone served as the normal control and those cultured with extracts containing equal volumes of normal saline served as the solvent control.

Cell viability was analyzed by CCK-8 staining and live/dead cell staining (Calcein-AM/PI), and the cell adhesion and viability were observed by fluorescence microscopy. For the live/dead cell staining, after continuous cultivation for 1, 2, and 3 days, an appropriate amount of Calcein-AM/PI mixed staining solution was added to each well. The cells were then incubated at 37 °C for 15 min, and the staining results and morphology of the cells were observed using a fluorescence microscope. The ImageJ software was used to analyze and calculate the number of live and dead cells.

Concurrently, the cells were also inoculated into a six-well plate using the same method, and the effect of the scaffold material on cell migration was determined by a cell wound healing assay. On the back of the six-hole plate, five horizontal lines were evenly drawn using a marker across the holes. The limbal stem cells were inoculated into the 6-well plate for 48 h, and the cells covered the plate. Then, a sterilized 200 μL pipette tip was cut perpendicular to the horizontal plane of the back, and the cells were washed 3 times with PBS solution to remove any dead cells. Complete medium containing 2% bovine serum albumin was added into the 4D-CTH cell carrier in the experimental group, but the control group was left untreated. Subsequently, scratch healing was detected at 0, 24, and 48 h, and cell mobility was calculated. The prepared 4D-CTH cell carrier was then placed under a UV lamp for 2 h. After sterilization, the carrier was placed into the culture plate and co-cultured with rLESCs. When the cells were covered with the carrier, the carrier carrying the cells was removed, fixed, dehydrated, and silver-plated. Then, it was observed and photographed by an SEM.

### Application and evaluation of 4D-CTH with encapsulated rLESCs for alkali burn wound healing

#### Construction of alkali burn models

Intraperitoneal injection of pentobarbital sodium for anesthesia in rats weighing 180–200 g (40 mg/kg body weight). The corneal model of a moderate alkali burn was established in the right eyes of the rats, while the left eyes were used as the control. Local anesthesia with 0.4% oxybupivacaine hydrochloride was performed on the ocular surface of the rats. Filter paper 3 mm in diameter was immersed in 1 mol/L NaOH solution for 1 min, then the filter paper was attached to the center of the cornea for 40 s. Then, the cornea was immediately rinsed with physiological saline for 1 min to establish an animal corneal alkali burn model [6]. After modeling for 15 days, rat corneas were isolated and sectioned into 6 μm frozen slices. These slices were then labeled with CK3/12 and CK13 to respectively mark corneal epithelial and conjunctival cells, in order to verify the effectiveness of the limbal stem cell deficiency (LSCD) model.

The experimental animals were randomly divided into normal, model, 4D-CTH–treated, traditionally treated and 4D-CTH carrier with encapsulated rLESCs (4D-CTH-rLESC) treated groups, with at least five animals in each group. In the 4D-CTH-rLESC treatment group, we collected rLESCs using cell basic culture medium at a concentration of 1 × 10^6^ cells per rat and prepared a cell suspension. We then loaded the rLESCs onto the printed 4D-CTH, allowing the rLESCs to be evenly distributed within the scaffold and the mesh. Before transplantation, the rLESCs will be incubated for 5 min. Subsequently, 4D-CTH-rLESC was applied immediately placed on the surface of the alkali-burned eye. For the traditional treatment group, CMCTS/SAD hydrogel was injected into the ocular surface with a custom-made double syringe, which was formed by connecting the ends of two needle tubes, the liquid pushed out from the two needle tubes will be mixed at the end and flow out through one needle. The two syringes were filled with sterile 2% CMCTS encapsulated with rLESCs (equivalent to the 4D-CTH-rLESC group) and sterile 2%SAD of the same volume, which were injected into the eye surface using the double syringe, and the CMCTS/SAD hydrogel quickly formed in situ under the control of the eyeball temperature [67]. The 4D-CTH group involved placing the printed 4D-CTH on the surface of the alkali-burned cornea and adding an equal amount of saline solution as the cell solution in the other two groups. The burned eye surface in the model group was washed with PBS. During treatment, the upper and lower eyelids of the rats were sutured, and levofloxacin eye drops were administered daily to prevent infection. Additionally, daily intraperitoneal injections of 5 mg/kg cyclosporine A (CyA) were given to suppress any potential rejection reactions from the allogeneic rLESCs transplantation. The normal rat group was not treated.

#### General observations of wound healing status

The stitches were removed from the eyeballs 3 days after the operation, and the eye surface and wound healing of each group were observed and photographed on days 0, 5, 10, and 15. According to the classification method of the American Academy of Ophthalmology (AAO), corneal neovascularization is divided into the following grades: Grade I: Neovessels grow at the corneal edge or surface, occupying less than 1/3. Grade II: Neovessels grow on the corneal surface, occupying 1/3 to 2/3. Grade III: Neovessels grow on the corneal surface, occupying more than 2/3. The cornea is divided into four quadrants, and the neovascularization in each quadrant is graded from 0 to 3. The total score for corneal neovascularization is then obtained by summing the scores from all four quadrants, resulting in a total score ranging from 0 to 12.

At the same time, the experimental eyes in each group were stained with 0.2 μL of sodium fluorescein and photographed with blue light. Then, the corneal epithelial photos from the different groups were analyzed using ImageJ software.

#### Histological observations and fluorescence microscope imaging

On the 15th day after surgery, the rats were euthanized, their eyeballs were removed, and the corneas were peeled off. The corneas were divided into two parts along the midline. One portion was fixed in 10% formalin, embedded in paraffin, stained with hematoxylin–eosin (HE), and finally observed under an optical microscope. The other half was placed in an optimal cutting temperature compound (OCT) embedded box, which was fixed in a refrigerator at − 80 °C to create frozen sections. After that, we placed the frozen OCT containing the rat corneas on a frozen slicer and prepared a 6 μm frozen section with a uniform thickness. Then, cytokeratin 3/12 and vimentin were stained with immunofluorescence and finally observed by a fluorescence microscope. The antibodies mentioned above were diluted 1:100 in 5% BSA as the diluent.

### Statistical analysis

SPSS v.19.0 software was used for statistical analysis of the experimental data, where the quantitative data were shown as mean ± standard deviation (x̄ ± SD). After confirming the normal distribution of the data, the mean difference was evaluated by a one-way ANOVA or *t* test, where *p* < 0.05 was considered to be statistically significant.

## Results

### 4D-CTH gelation and microstructure observations

**Fig. 1:**
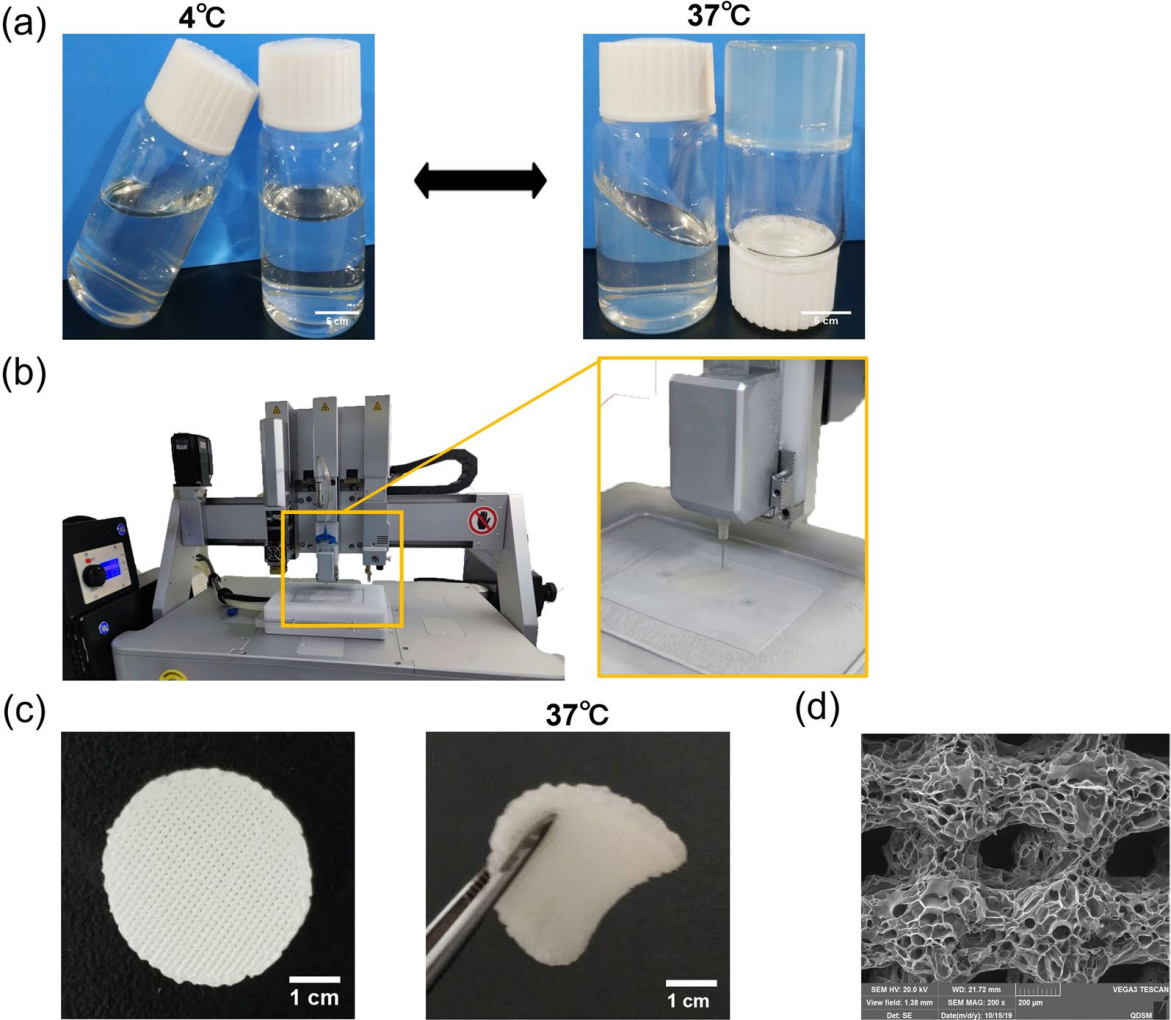
Synthesis and properties of 4D-CTH. **a** The thermoreversible sol–gel transition of the CTH between 4 and 37 °C. Scale bar = 5 cm. **b** The preparation process of 4D-CTH. **c** The structure of CTH prepared via 4D printing (left). 4D-CTH became gelatinous at 37 °C (right). Scale bar = 1 cm. **d** Electron micrograph of 4D-CTH. Scale bar = 200 μm

The prepared CTH exhibited a flowing sol state at 4 °C and solidified gel state when the temperature increased to about 37 °C (Fig. 1a). We used a self-modified biological printer to prepare 4D-CTH that could be deformed in response to temperature changes. According to the measured rat corneal data, a three-dimensional model of 4D-CTH was constructed by matching software, and the temperature-sensitive CTH was injected into the biological printer to print the 4D-CTH which could become gel at 37 °C under the control of low temperature and specific parameters (Fig. 1b). The appearance of the 4D-CTH was white with a porous structure and uniform thickness (Fig. 1c). SEM analysis showed that the scaffold material had regularly shaped pores and interconnected internal structures (Fig. 1d).

### Physical behavior of 4D-CTH

**Fig. 2:**
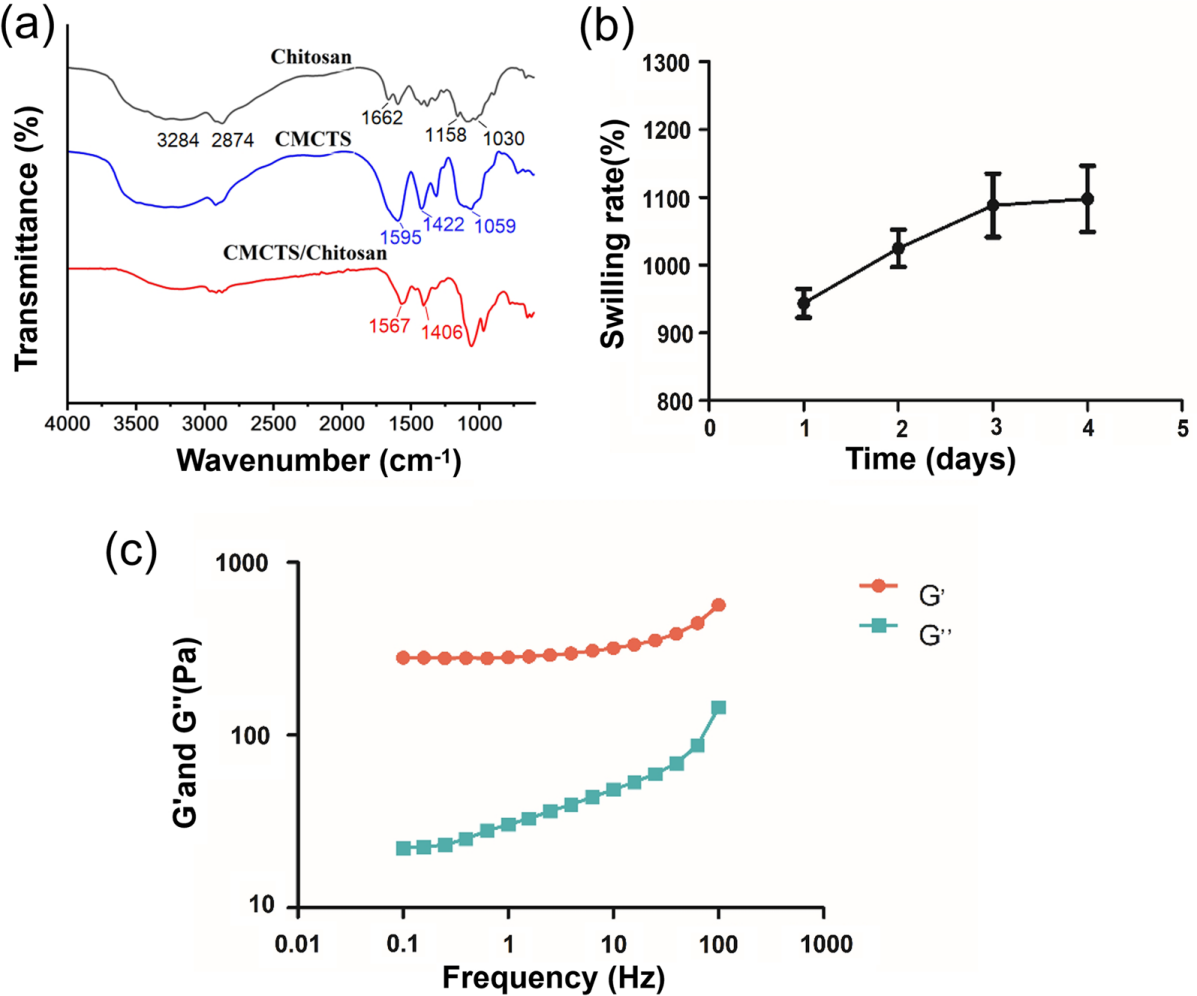
Physical behavior of 4D-CTH. **a** Analysis of chitosan, CMCTS and chitosan-based thermosensitive gel (CMCTS/Chitosan) by ATR-FTIR. **b** Swelling rate of 4D-CTH cell carrier. **c** Storage modulus and loss modulus of chitosan-based temperature-sensitive gel measured in the frequency range of 0.1–100 Hz

The FTIR spectra of chitosan, CMCTS and their cross-linked chitosan-based thermosensitive hydrogel (CTH) are shown in Fig. 2a. The absorption peaks of chitosan at around 3284 and 2874 cm^−1^ correspond to the stretching vibrations of O–H and C–H bond respectively [57], the peak at 1662 cm^−1^ is bending vibration of N–H bond, and the asymmetric bridge O stretching and C–O stretching at 1158 and 1030 cm^−1^ respectively [28]. The absorption peaks of CMCTS at 1595 and 1422 cm^−1^ correspond to the asymmetric and symmetrical stretching vibrations of carboxyl groups, respectively, which proves the existence of carboxyl groups [57]. In addition, the C–O–C characteristic peak at 1059 cm^−1^ also confirmed the formation of CMCTS [62]. After mixed with β-glycerophosphoric disodium and chitosan, the absorption peak of CMCTS at 1595 and 1422 cm^−1^ shifts to low wavenumber, which may be due to the cross-linking of CTH [13].

The swelling rate of the 4D-CTH cell carrier is shown in Fig. 2b, indicating that the carrier scaffold reached swelling equilibrium after it was immersed in double-distilled water for four days. On the fourth day, the swelling rate was 1097.54 ± 84.46% (Fig. 2b). The viscoelastic behavior of the chitosan-based thermosensitive gel was measured by dynamic oscillation rheology, and the function diagrams of the storage modulus (G′) and loss modulus (G″) as a function of frequency were drawn (Fig. 2c). The results showed that the storage modulus and loss modulus of the chitosan-based thermosensitive gel increased with increasing frequency in the frequency range of 0.1–100 Hz, and the storage modulus was always greater than the loss modulus. As discussed in the previous sections of swelling ratio and viscoelastic behavior measurement, the results of mechanical properties indicating that the prepared carrier had excellent hydrogel properties [22].

### Acquisition, primary culturing, and identification of the rat limbal stem cells

**Fig. 3:**
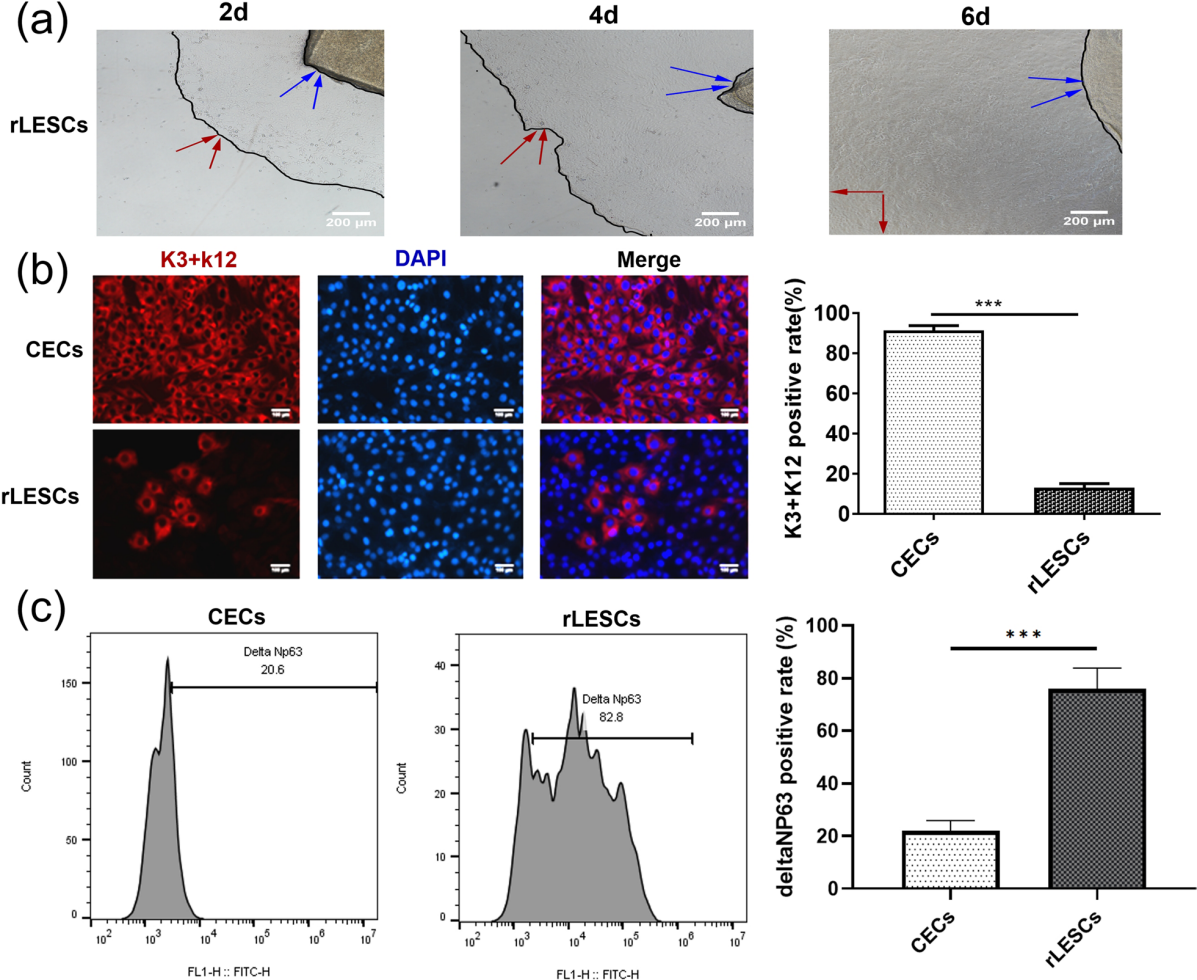
Primary culture, immunofluorescence and flow cytometry identification of rLESCs. **a** Representative images (40×) of rLESCs migration on day 2, 4, and 6. The blue arrows indicate the edge of the tissue block. The red arrows indicate the edge of the migrated rLESCs. **b** Representative images of cytokeratin 3/12-stained CECs and rLESCs (left) and the corresponding positive rates in CECs and rLESCs (right). Scale bars = 100 μm. **c** Representative flow cytometrical detection of deltaNP63 expression (left) and the corresponding positive rates (right) in CECs and rLESCs. The isotype control images for the rLESCs group are presented in Additional file 1: Figure S1. Data is presented as the mean ± SD for each group from the three experiments. ***p < 0.001

As described in the methods section, the rLESCs were obtained by tissue mass culturing in rats. As shown in Fig. 3a, we judged whether the cells proliferated and migrated by observing the moving distance that intersected with the boundary of the adherent cells the day before under an inverted microscope. On the second day, most cells migrated around the tissue mass (Fig. 3a), while on the fourth day, some cells were removed from the tissue mass (Fig. 3a). On the sixth day, the cells around the tissue mass fused and jointed tightly, growing like paving stones (Fig. 3a). Next, the rLESCs were identified by cell immunofluorescence staining and flow cytometry. Corneal epithelial cells (CECs) were used as the positive or negative control for cytokeratin 3 and 12 and deltaNp63 expression, respectively. The results showed that the positive rate of cytokeratin 3 and 12 in the rLESCs was less than 20% (Fig. 3b) (***p < 0.001), while the positive rate of the deltaNp63 protein was 75.97 ± 7.911% (Fig. 3c) (***p < 0.001) in the ex vivo expanded rLESCs. These results indicated that our primary cultured cells consisted of a mixed epithelial cell population that was mainly composed of rLESCs, which could also be called rLESC-enriched limbal epithelium.

### 4D-CTH cytocompatibility with the rLESC-enriched limbal epithelium in vitro

**Fig. 4:**
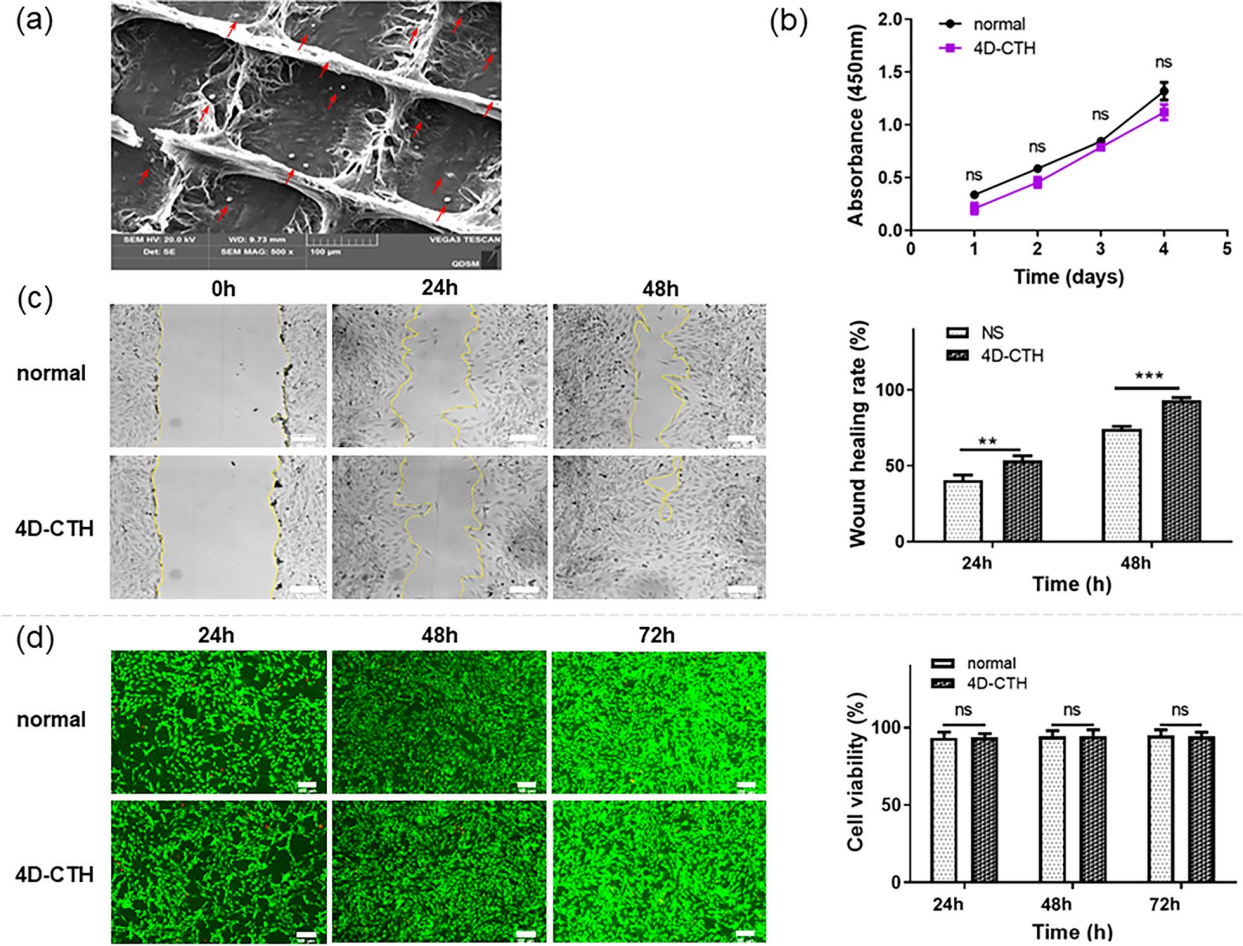
Cytocompatibility of 4D-CTH cell carriers. **a** After 4D-CTH carrier was dried, the transferred cells were observed by electron microscope. The red arrows indicate the rLESCs adhered to the scaffold and partially distributed within the mesh. **b** CCK-8 analysis of rLESC-enriched limbal epithelium seeded in the normal culture medium and scaffold material (n = 3). **c** Representative images (left) and quantitative analysis of the wound healing rate (right) of the scratch area in the control group and scaffold material group at 24 and 48 h. **d** Representative live/dead images (left) and quantification of cell viability (right) in the normal culture medium and scaffold material after 24, 48, and 72 h of culturing. The data is expressed as mean ± SD **p < 0.01; ***p < 0.001, n ≥ 3

The rLESC-enriched limbal epithelium were inoculated on a 4D-CTH cell carrier, cultured for 48 h and observed by SEM. The results showed that the cells were closely connected and grew in pieces (Fig. 4a). Cytotoxicity and cell scratch tests were used to determine the cytocompatibility of the prepared 4D-CTH cell carriers. The CCK-8 assay results indicated that there was no significant difference in cell proliferation between the normal and 4D-CTH groups (*p* > 0.05). These results suggested that the scaffold material did not inhibit the proliferation of the cell population (Fig. 4b). The cell scratch test results also showed that the wound healing rates of the 4D-CTH group were 53.49 ± 3.058% and 93.28 ± 1.777% at 24 and 48 h, respectively. These rates were significantly higher than the control group at the same time periods (Fig. 4c), indicating that the scaffold material could promote the migration of rLESC-enriched limbal epithelium. The live/dead cell staining results showed that the cell survival rates in both groups were higher than 90%, and the cells also showed a typical rLESC cell morphology (Fig. 4d), indicating that the 4D-CTH cell vector could provide a suitable environment for cell growth.

### Treatment of ocular alkaline burn wounds using 4D-CTH encapsulated with rLESC-enriched limbal epithelium

#### General observation of wound healing status

**Fig. 5:**
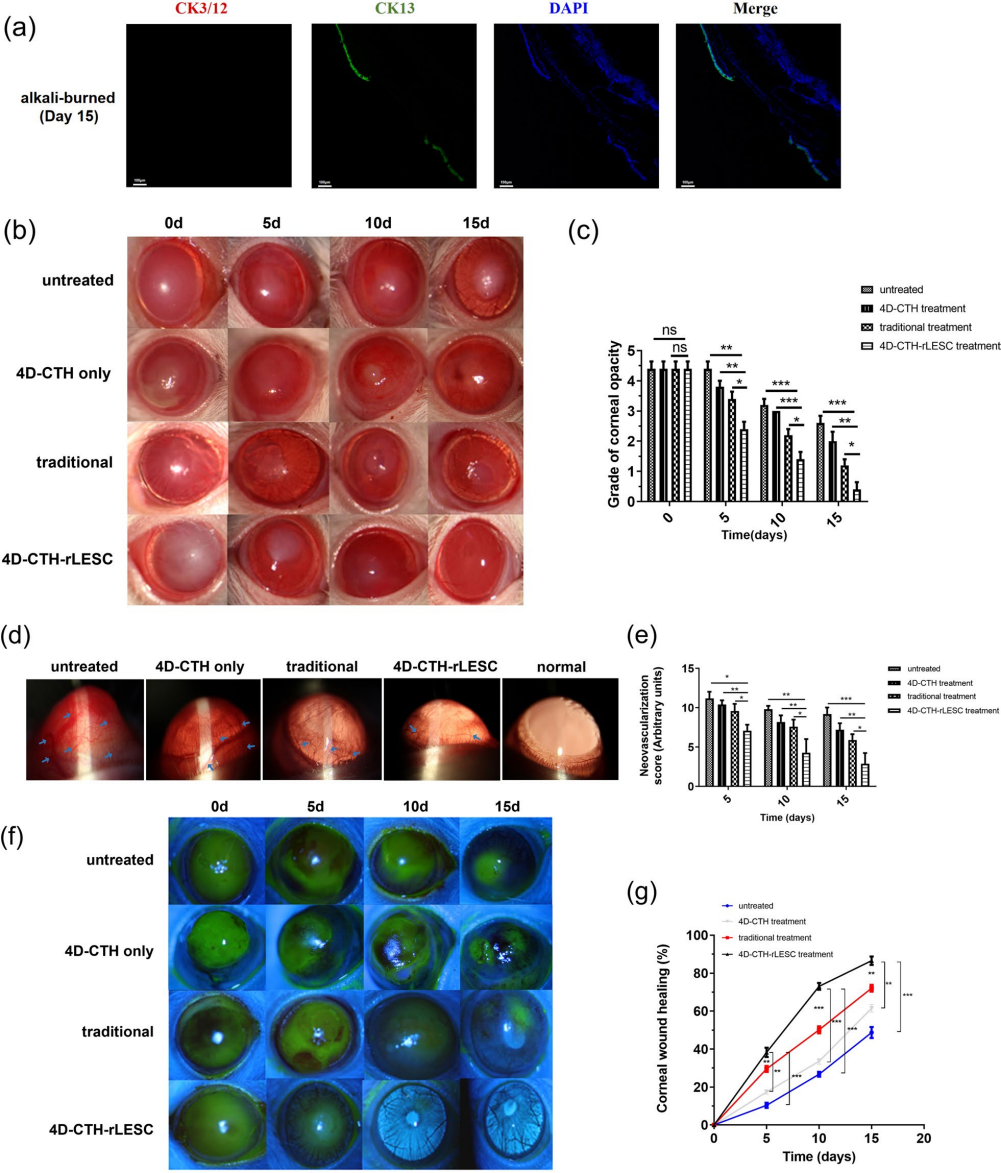
General observation of wound healing status after treatment of ocular alkaline burn wound by 4D-CTH encapsulating rLESC-enriched limbal epithelium. **a** Immunofluorescent staining results of CK3/12 and CK13 in the rat corneal alkali burn model at day 15. Cytokeratin 3 and 12 (red) and Cytokeratin13 (green) were used as markers of the epithelium and conjunctiva. Scale bars = 100 μm. **b** Representative images of corneal opacity in the untreated alkali burned, 4D-CTH treated, traditionally treated, and 4D-CTH-rLESC treated groups at 0, 5, 10, and 15 days post-burn. **c** Corneal opacity score results. **d** Representative images of corneal neovascularization in the untreated alkali burned, 4D-CTH treated, traditionally treated, 4D-CTH-rLESC treated and normal groups at 15 days post-burn. **e** Corneal neovascularization score results. **f** Representative images of damaged corneal epithelium stained with sodium fluorescein in the untreated alkali burned, 4D-CTH treated, traditionally treated, and 4D-CTH-rLESC treated groups at 0, 5, 10, and 15 days post-burn. **g** Results of corneal epithelial wound healing level. The data expressed as mean ± SD of five rats per group. *p < 0.05, **p < 0.01, ***p < 0.001, n = 5

Corneal alkali burn models in rats were created and the effects of stem cell transfer by different carriers were observed. The results of immunofluorescence staining showed that the epithelial cells around the remaining epithelial wound were negative for CK3/12 and positive for CK13 (conjunctival marker) (Fig. 5a). This indicates the effectiveness of the LSCD model, with the corneal epithelium being replaced by conjunctival epithelium in the untreated group (Fig. 5a). The control group was divided into the 4D-CTH-only treatment group, the traditional treatment (our previously developed material-ACH) group and the 4D-CTH-rLESC treatment group, and evaluated the therapeutic effect of the two treatments by observing the corneal recovery of different groups of rats. At the same time, the 4D-CTH-rLESC group was compared with the traditional treatment group and the normal group to evaluate whether the efficacy of the 4D-CTH-rLESC was better than that of the traditional treatment (ACH-rLESC), and whether the repair effect could be close to the normal rat cornea. The evaluation index of corneal opacity and neovascularization after alkali burn in rats were based on previous study [32]. Compared to the untreated model, the 4D-CTH scaffold only and traditionally treated groups, the 4D-CTH-encapsulated rLESC-enriched limbal epithelium significantly reduced the corneal opacity score and inhibited neovascularization (Fig. 5b–e) (*p* < 0.01 or *p* < 0.05). In addition, fluorescein sodium staining showed that compared to the model and traditional treated groups, the treatment of the 4D-CTH-encapsulated rLESC-enriched limbal epithelium significantly accelerated the healing process of the corneal wounds (Fig. 5f, g) (*p* < 0.01 or *p* < 0.001), and the corneal epithelial wound healing level reached 86.60 ± 5.004% on the 15th day. Additionally, the corneal opacity, neovascularization, and the repair of damaged corneal epithelium in rats at 30 and 60 days were similar to those observed at 15 days (Additional file 1: Figure S2).

#### Histological analysis of the wounded cornea

**Fig. 6:**
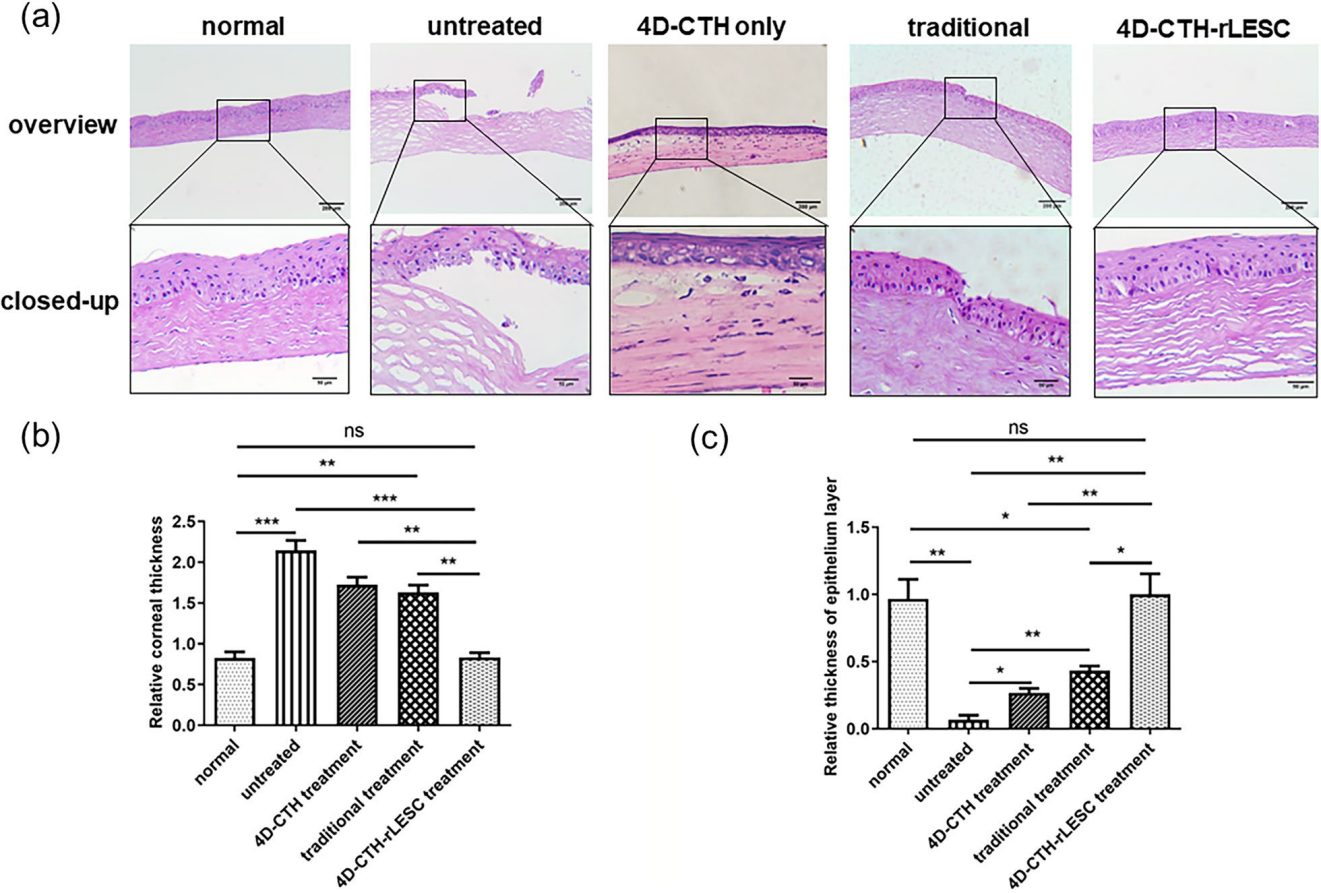
Histological analysis results of the wounded cornea after treatment of ocular alkaline burn wound by 4D-CTH encapsulating rLESC-enriched limbal epithelium. **a** Representative images of corneal histology in the normal group, model group, 4D-CTH only group, traditionally treated group and 4D-CTH-rLESC treated group at 15 days post-burn. Scale bars = 200 μm (overview), 50 μm (closed-up). Corneal thickness (**b**) and relative thickness of corneal epithelial layer (**c**) in the normal group, model group, 4D-CTH only group, traditionally treated group and 4D-CTH-rLESC treated group at 15 days post-burn. The red arrows indicate the goblet cells in the conjunctivalized area of the untreated group. Data is presented as the mean ± SD for each group, *p < 0.05; **p < 0.01; ***p < 0.001, n ≥ 3

The 4 cornea groups were collected for tissue sectioning and HE staining on day 15 post-burn. The results showed that the normal corneal tissue structure was clear and complete, including the epithelial, stroma, and endodermis layers (Fig. 6a). In the untreated model group, the cornea had obviously thickened with an unclear corneal structure, broken epithelial layer, and obvious defect areas (Fig. 6a). In the traditionally treated group, the cornea had also significantly thickened, with a slight fracture in the epithelial layer. In the 4D-CTH-rLESC treated group, the corneal structure was intact, with all layers clearly visible, and a complete, continuous, smooth epithelial layer (Fig. 6a). After 15 days, the corneas in the 4D-CTH-rLESC group were similar to the corneas in the normal group, and had a more complete corneal structure than the other 2 groups. In addition, the corneal and epithelial thicknesses of each group were analyzed using ImageJ software. Compared to the normal unburned group, the corneal thickness of the 4D-CTH-rLESC group showed no significant changes (*p* > 0.05), while the thicknesses of the model and traditionally treated groups increased significantly (*p* < 0.05) (Fig. 6b). In the corneal epithelial layer, there was no significant difference between the 4D-CTH-rLESC treated and normal groups; however, in the traditionally treated and model groups, the epithelial layer was significantly thinner or even missing (Fig. 6c). Therefore, after treatment of the 4D-CTH-encapsulated rLESC-enriched limbal epithelium, the corneal stroma and epithelial layer largely recovered to normal, and their thicknesses were similar to the normal group. The treatment effect of the 4D-CTH-rLESC group was significantly better than that of the other three groups.

#### Immunofluorescence analysis of wound healing status

**Fig. 7:**
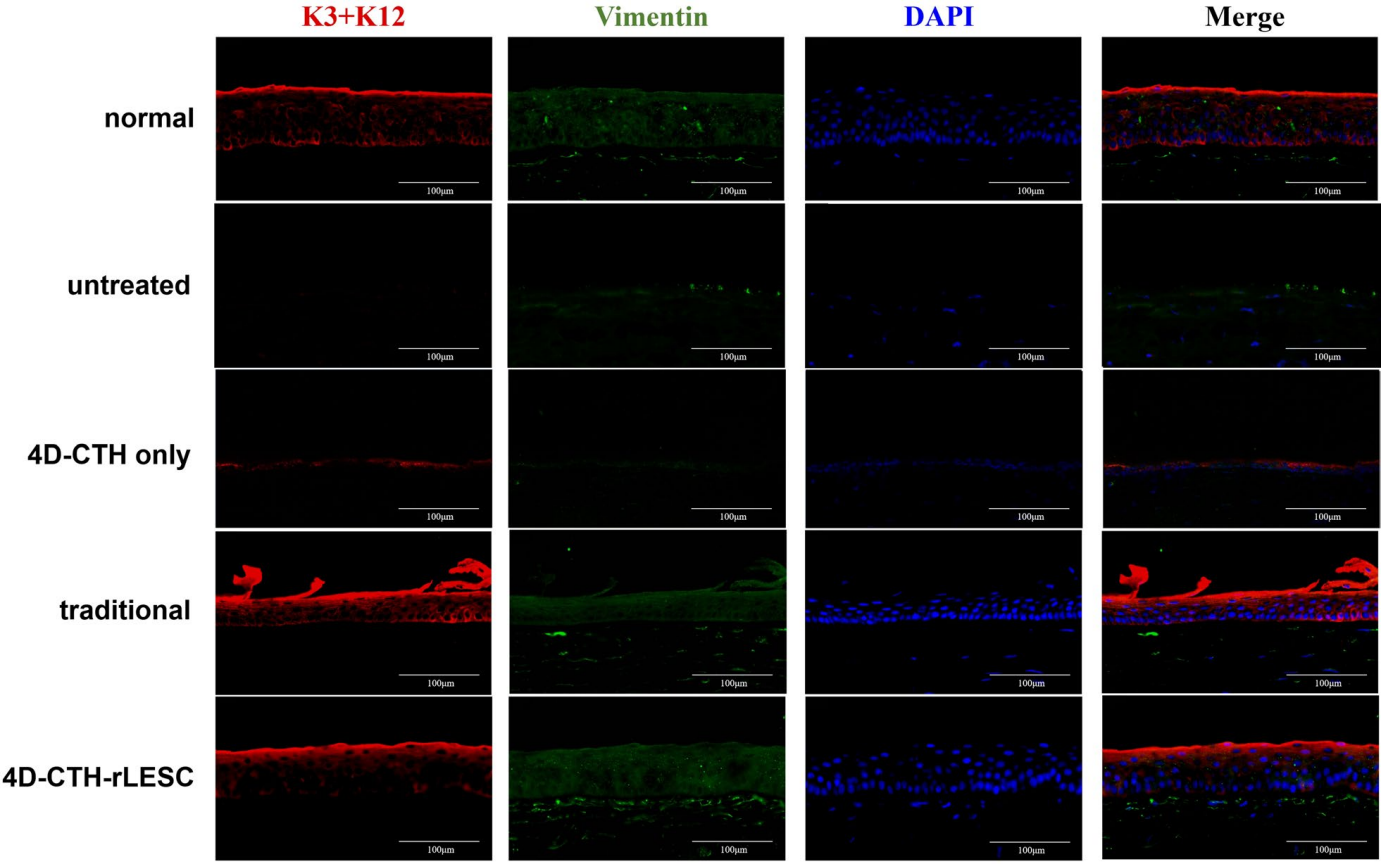
Immunofluorescence analysis results of wound healing status after treatment of ocular alkaline burn wound with 4D-CTH encapsulating rLESC-enriched limbal epithelium. Cytokeratin 3 and 12 (red) and vimentin (green) were used as markers of the epithelial and stromal cells, respectively, in the unburned (normal), untreated (model), 4D-CTH treated, traditionally treated, and 4D-CTH-rLESC treated alkali burned rat corneas. Scale bars = 100 μm

The corneas of the rats were collected 15 days after alkali burning, and immunofluorescence staining was performed on the frozen tissue sections. Cytokeratin 3/12 was used as the marker protein in the corneal epithelial layer, and vimentin protein was used as the marker protein in the corneal stroma layer. The results showed continuous expression of cytokeratin 3 and 12 in the corneal epithelial layer of the 4D-CTH-rLESC treated group and traditional group, which were similar to the unburned group (Fig. 7), and the fluorescence signal of CK3/CK12 in the 4D-CTH-rLESC group is strong and continuous (Additional file 1: Figure S3). For the model groups, cytokeratin 3 and 12 expression was not continuous, and the fluorescence signal intensity of CK3/12 was weaker in the 4D-CTH-only treated group (Fig. 7). However, uneven and protuberant corneal epithelium can be observed in some traditional treatment groups, while the corneal epithelial structure of the 4D-CTH-rLESC group is more complete and smooth. In a word, the repair efficiency of rLESCs encapsulated by 4D-CTH is better than that of the 4D-CTH only and the traditional treatment group.

## Discussion

Severe corneal alkali burn can cause corneal epithelial adhesion and even corneal blindness, and the prognosis is poor [27]. An intact corneal epithelium can hinder the invasion and injury of pathogenic microorganisms, as well as inhibit immune inflammation and corneal neovascularization [34, 65]. LESCs are an important source of epithelial regeneration [60]. Alexander et al. indicated that corneal epithelial healing was closely related to the regeneration of limbal stem cells and reconstruction of the basement membrane [38]. During corneal wound healing, limbal epithelial stem cells migrated to the center of the cornea, proliferated rapidly, and differentiated into corneal epithelial cells [23, 46, 55]. To solve the problem of cell regeneration after corneal injury, researchers have attempted to culture autologous or allogeneic LESCs in vitro for corneal transplantation [4, 14, 49], as LESCs have many advantages, including accessibility and low immunogenicity [17, 50]. However, even so, allogeneic LESC transplantation still carries the risk of rejection reactions that can significantly impact the therapeutic outcome [3, 51]. While autologous limbal stem cell transplantation has a higher success rate, it is only effective for unilateral or partially bilateral diseases and is not suitable for all bilateral diseases [5]. Therefore, it is necessary to explore methods for allogeneic limbal stem cell transplantation. Existing studies indicate that adequate immunosuppression following transplantation is crucial for the effectiveness of allogeneic LESC transplantation and the restoration of visual acuity in transplant recipients [5, 26, 45]. In this study, we chose to use allogeneic rat-cultured limbal epithelial stem cells for the treatment of severe alkali-burned rat corneas under the premise of immunosuppression, in order to explore a more effective corneal epithelial stem cell transplantation strategy.

Many preclinical studies have confirmed that collagen-based hydrogels can promote the regeneration of damaged cornea [40, 44]. However, these hydrogels have poor mechanical properties and will easily degrade, limiting their applications as scaffolds for cell transplantation [7, 30, 40]. In our previous study, we confirmed that the transfer of LESCs to alkali-burned rabbits using an in situ hydrogel significantly promoted the repair of the injured ocular surface [67]. However, SEM analysis showed that the internal aperture of the hydrogel was different, which affected the therapeutic effect.

Although based on 3D printing technology, 4D printing technology has one more time dimension than 3D printing. A smart material with a shape memory function can be printed into a preset structure using 3D printing, which can then be used to produce expected shape changes when stimulated by external conditions [8, 66]. 4D printed shape-memory materials have obvious advantages. They can not only result in simple shape changes, but also realize many functions by presetting the deformation scheme, such as self-deformation, self-assembly, and self-repair [24, 41, 59, 68]. Research on 4D bioprinting for tissue engineering has been increasing. For example, Miao et al. used a 3D photocurable printer and a new, renewable soybean oil epoxidized acrylate material to produce a material that could automatically deform with changes in temperature. This material promoted the growth, adhesion, and proliferation of human bone marrow mesenchymal stem cells [21, 42, 43]. Hendrikson et al. prepared cell scaffolds with two fiber arrangement directions and a shape memory function by combining 4D printing technology with a polyurethane biomaterial, and the study confirmed that mechanical stimulation caused by the deformation of cell scaffolds led to the directional growth of cells and nuclei [21]. Miao et al. synthesized a new shape memory polymer through the chemical crosslinking of castor oil, polycaprolactone triol, and polyisocyanate, while polylactic acid was used to prepare the tissue scaffolds with a shape memory function and high biocompatibility via 4D printing. The scaffolds also had a biomimetic gradient gap structure that changed over time, which not only accommodated tissue cell growth, but also delivered necessary nutrients for cell growth and excreted waste generated by the cells [42, 43]. Overall, 4D-printed biomaterials exhibit many excellent properties and have shown great potential.

However, there have been few studies attempting to transplant corneal limbal stem cells, and no one has used this particular type of cell carrier to treat severe corneal alkali burns. Therefore, this study attempts to introduce 4D printing technology to prepare chitosan-based thermosensitive hydrogel carriers with uniform pore size through 4D bioprinting, in order to transplant stem cells to the site of corneal injury, providing essential seed cells for tissue repair. A series of experiments were conducted to comprehensively evaluate various performance indicators of the carriers.

In this study, 4D bioprinting was employed to enhance the performance of cell carriers, further improving cell loading efficiency and repair effects. It was found that compared with traditional 3D bioprinting, the 4D-CTH can change shape with temperature variation, reverting to a soft hydrogel state at 37 °C, which better aligns with the dynamic nature of biological tissue (Fig. 1c). Scanning electron microscopy observation of the microstructure of the carrier surface revealed uniform pore size and even cell distribution, which is of great significance for increasing cell loading rates and cell density (Figs. 1d, 4a). In addition, we characterized the physical properties of 4D-CTH, including ATR-FTIR, swelling ratio determination, and viscoelasticity testing, and found that the carrier exhibited excellent performance, meeting the requirements of tissue engineering carrier materials.

Previous studies have indicated that during the process of corneal wound healing, limbal epithelial stem cells (LESCs) undergo proliferation, differentiate into corneal epithelial cells, and migrate centripetally, thereby promoting injury repair [69]. The isolation and identification of LESCs rely on the specific biological marker DeltaNp63, which is highly expressed in corneal limbal epithelial cells and serves as a crucial regulatory factor for corneal epithelial cell differentiation and proliferation, widely recognized as a marker for LESCs [19, 25, 48]. In this study, immunofluorescent staining of the corneal epithelial markers CK3 and CK12 was conducted, and flow cytometric analysis of the characteristic protein DeltaNp63 in rat LESCs (rLESCs) was performed to identify the extracted primary cells, ensuring the purity of the transplanted cells (Fig. 3b, c).

Great cell compatibility is a crucial characteristic required for an excellent corneal tissue engineering scaffold. Through electron microscopy observation, it was found that rLESCs could evenly adhere to the dried 4D-CTH (Fig. 4a). CCK8 analysis and live/dead cell staining results indicated that the cell viability of LESCs grown in the culture medium with added carrier material showed no significant difference compared to that in the general cell culture medium (Fig. 4b, d). Furthermore, we observed a slightly increased migration rate of rLESCs and a faster scratch healing rate in the presence of the carrier material (Fig. 4c), indicating that 4D-CTH could provide a better proliferation environment for rLESCs.

Chitosan and its derivatives have been demonstrated to promote the repair of corneal epithelium [16, 54]. Previous studies have indicated that CMCTS decreased abdominal adhesions and reduced fibrosis, which is crucial for inhibiting scar formation and maintaining epithelial integrity [35, 37, 54]. In our previous studies, we applied a chitosan-based in situ hydrogel (ACH) to transplant stem cells onto alkali-burned eyes, significantly accelerating the healing process of rabbit corneas [67]. However, issues such as low cell delivery efficiency and unsatisfactory repair outcomes were observed. In this study, we evaluated the efficiency of a novel stem cell carrier by assessing the reparative effects of alkali-burned corneas in SD rats. We used 4D-CTH to deliver rLESC to the ocular surface of alkali-burned rats and compared this with the traditional treatment group, in which rLESC were embedded in ACH. We regularly observed the corneal transparency, neovascularization, and wound healing rate of the different treatment groups. The results showed that 15 days after treatment, the corneal transparency of rats in the 4D-CTH-rLESC group significantly improved, with a marked decrease in opacity scores (Fig. 5b, c), reduced neovascularization (Fig. 5d, e), significantly improved corneal epithelial healing (Fig. 5f, g), and noticeable improvement in epithelial morphology (Figs. 6, 7).

In summary, the transplantation of 4D-CTH-rLESC significantly improves corneal alkali burn wound healing, with a therapeutic effect superior to that of the carrier-only treatment group and the traditional treatment group. It is exciting to note that the neovascularization score in the 4D-CTH-rLESC group was lower compared to the traditional treatment group, which may be associated with the inhibitory effect of 4D-CTH on local corneal inflammation following alkali burns. Furthermore, this inhibitory effect may be stronger than that of ACH, as suggested by our previous studies. In future research, we will further investigate the mechanism by which this novel cell carrier inhibits neovascularization and promotes corneal epithelial repair. Large animal experiments involving pigs, dogs, and monkeys will be conducted to expedite the clinical translation of these findings.

In this study, a limbal stem cell carrier with a uniform aperture and adjustable shape was prepared using natural marine extracts and 4D printing technology, and the performance indices of the carrier were comprehensively evaluated through a series of experiments. The results showed that the performance index of the vector was superior to the traditional vector, and significantly improved the cell transfer efficiency and the effect of corneal wound repair after alkali burn. During corneal wound healing, the LESCs proliferated and differentiated into corneal epithelial cells and migrated to the heart, which has been shown as the key to wound healing [18, 69]. Animal experiments confirmed that the rLESC-enriched limbal epithelium transplanted with this vector significantly promoted wound healing, while HE staining and immunofluorescence staining showed that the repair effect of the rLESC-enriched limbal epithelium was significantly improved compared to the traditional treatment group. In addition to repairing corneal alkali burns, the 4D-CTH vector could also repair other tissue damage, such as skin, diabetic foot, and cervical erosion injuries, offering broad application prospects in the future.

## Conclusions

The 4D-CTH cell scaffold prepared in this study showed good cell compatibility, a regular pore structure, and uniform cell distribution, which significantly improved the cell transfer capacity. Overall, this study confirmed that the 4D-CTH-encapsulated rLESCs could significantly improve the corneal repair effect in rats after alkali burning, accelerating the reconstruction of injured epithelium tissue. In conclusion, the prepared 4D-CTH may become an effective scaffold for cell transplantation and promote the repair of injured tissues.

In this study, natural marine extracts were used to prepare LESCs carriers using 4D bio-printing technology, overcoming the bottleneck issue of different pore sizes and low degree of fit on the wound surfaces of traditional carriers. Subsequently, it offered good cellular compatibility and significantly improved the transshipment and local survival rates. Animal experiments confirmed that the 4D-CTH-encapsulated rLESCs significantly improved the corneal repair effect in rats with alkali burns and accelerated the reconstruction of injured epithelial tissues, indicating broad application prospects in the future.

### Supplementary Information

**Additional file 1. Fig S1.** The corresponding isotype control image for the rLESC group in Fig. 3c. The isotype control antibody was rabbit IgG (Abcam, AB172730). **Fig S2.** Representative images of the corneal opacity, neovascularization, and the repair of damaged corneal epithelium in 4D-CTH-rLESC group at 30 and 60 days. **Fig S3.** Representative immunofluorescence images of corneal epithelium labeled with CK3/CK12 observed under low magnification in 4D-CTH-rLESC group. Cytokeratin 3 and 12 (red) were used as markers of the epithelium. Scale bars =500 μm.

## Abbreviations

3D: Three-dimensional
4D: Four-dimensional
4D-CTH: Chitosan-based thermosensitive gel carrier using 4D bioprinting technology
4D-CTH-rLESC: 4D-CTH carrier with encapsulated rLESCs
AAO: American Academy of Ophthalmology
ACH: Alginate-chitosan hydrogel
ATR-FTIR: Attenuated Total Reflection Flourier transformed Infrared Spectroscopy
CCK-8: Cell Counting Kit-8
CECs: Corneal epithelial cells
CTH: Chitosan-based thermosensitive hydrogel
ECM: Extracellular matrix
GAG: Glycosaminoglycans
HCECs: Human corneal epithelial cells
HE: Hematoxylin–eosin
LESCs: Limbal epithelium stem cells
OCT: Optimal cutting temperature compound
PBS: Phosphate buffer solution
rLESCs: Rat limbal epithelial stem cells
SD rats: Sprague Dawley rats
