# Supplementary material for: The application of a 4D-printed chitosan-based stem cell carrier for the repair of corneal alkali burns
Source: Stem Cell Res Ther. 2024 Feb 14;15:41. doi: 10.1186/s13287-024-03653-z (PMC10865625; doi:10.1186/s13287-024-03653-z)
Supplement: Supplementary file 1 — Additional file 1. Fig S1. The corresponding isotype control image for the rLESC group in Fig. 3c. The isotype control antibody was rabbit IgG (Abcam, AB172730). Fig S2. Representative images of the corneal opacity, neovascularization, and the repair of damaged corneal epithelium in 4D-CTH-rLESC group at 30 and 60 days. Fig S3. Representative immunofluorescence images of corneal epithelium labeled with CK3/CK12 observed under low magnification in 4D-CTH-rLESC group. Cytokeratin 3 and 12 (red) were used as markers of the epithelium. Scale bars =500 μm. [file 13287_2024_3653_MOESM1_ESM.docx]

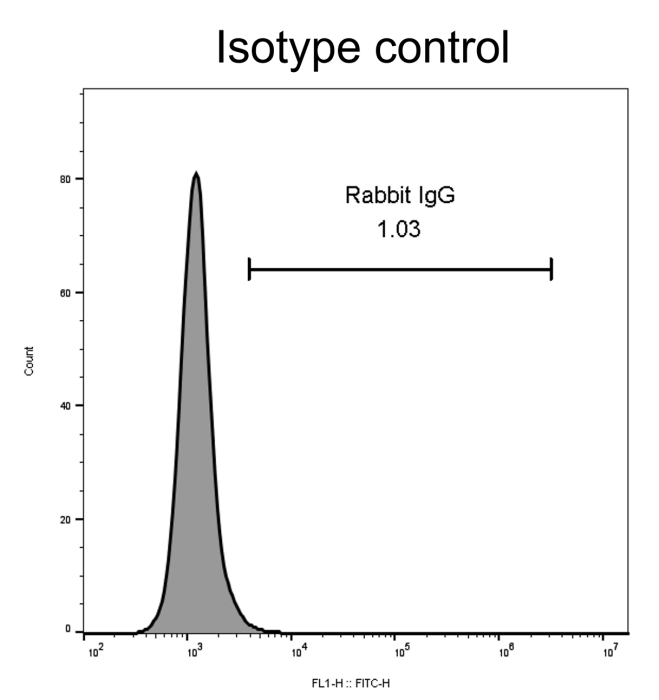


**Fig S1. The corresponding isotype control image for the rLESC group in Fig. 3c. The isotype control antibody was rabbit IgG (Abcam, AB172730).**


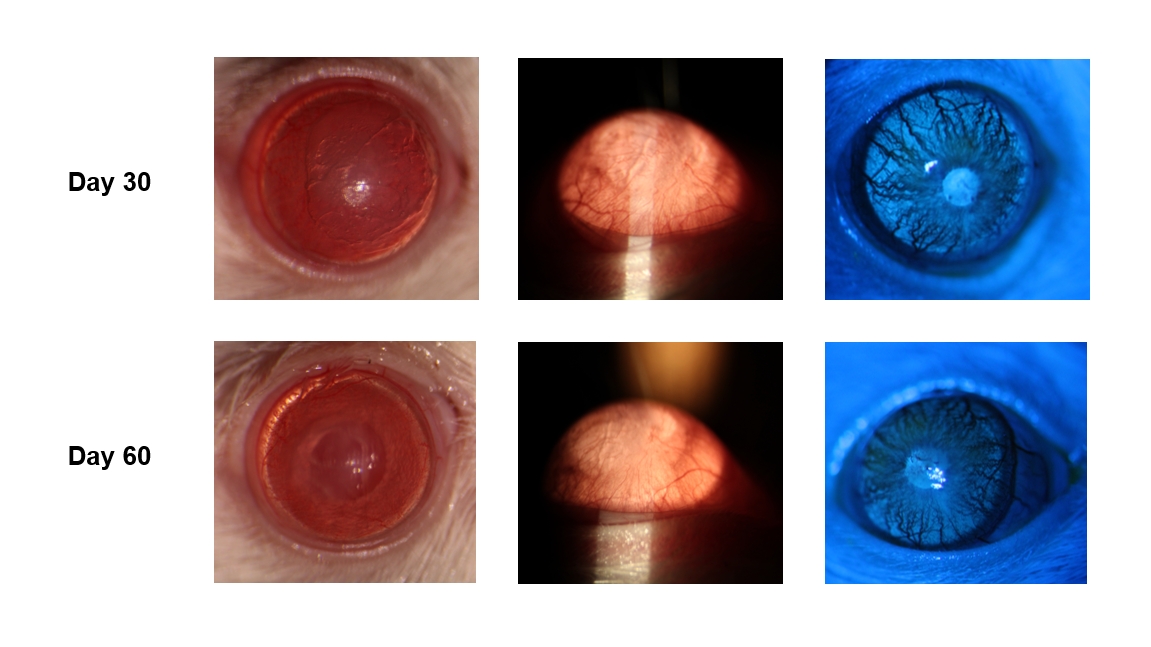


**Fig S2. Representative images of the corneal opacity, neovascularization, and the repair of damaged corneal epithelium in 4D-CTH-rLESC group at 30 and 60 days**


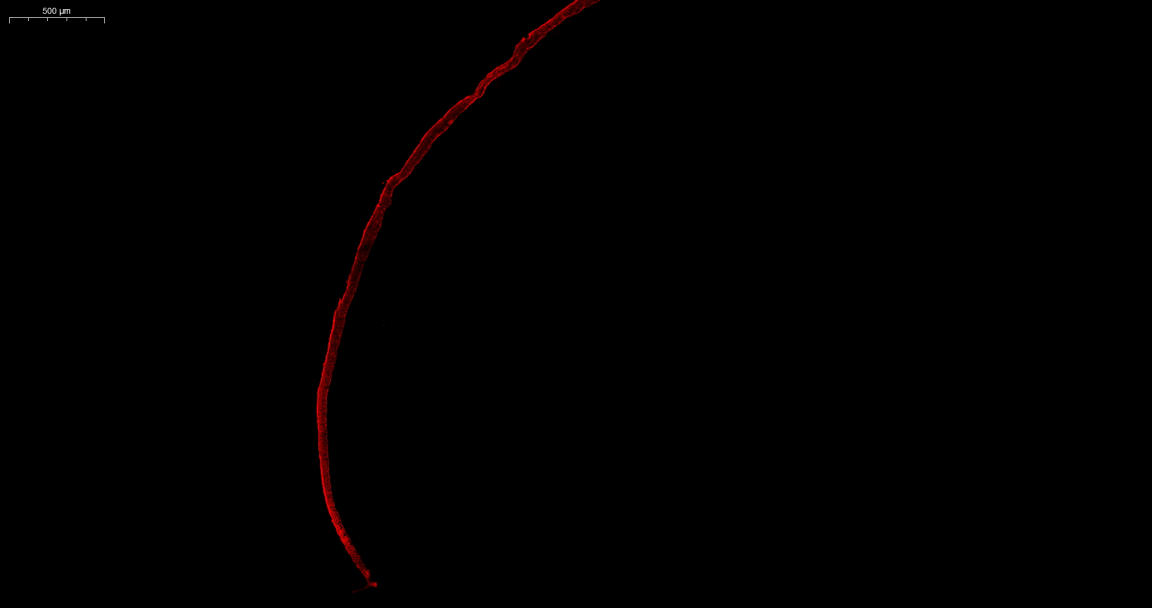


**Fig S3. Representative immunofluorescence images of corneal epithelium labeled with CK3/CK12 observed under low magnification in 4D-CTH-rLESC group.**Cytokeratin 3 and 12 (red) were used as markers of the epithelium. Scale bars =500 μm
